# Does instrumentation and irrigation configuration affect intrarenal pressure during PCNL?

**DOI:** 10.1002/bco2.70164

**Published:** 2026-02-04

**Authors:** Evan Seibly, Ali Albaghli, Kyu Park, Elizabeth A. Baldwin, Ala'a Farkouh, Katya Hanessian, Nicole Mack, Cliff De Guzman, Toby Clark, Matthew Buell, Rose Leu, Kanha Shete, Sikai Song, Akin S. Amasyali, Zham Okhunov, D. Duane Baldwin

**Affiliations:** ^1^ Department of Urology Loma Linda University Health Loma Linda California USA

**Keywords:** **i**nstrumentation, lithotripsy, nephrolithiasis, percutaneous nephrolithotomy, pressure

## Abstract

**Objectives:**

To measure the effects of varying configurations of nephroscope sheath, irrigation, instruments, and suction on intrarenal pressure (IRP) during percutaneous nephrolithotomy (PCNL).

**Materials and Methods:**

Kidney and ureter 3D printed models from a deidentified patient's CT scan were placed in a plaster and foam mould, simulating the torso of a prone patient. An Amplatz sheath was inserted into the kidney model. Fourteen different rigid nephroscope sheath (RNS), irrigation, instrument and suction configurations were compared. IRP was measured in a retrograde fashion. Comparisons were performed using the Wilcoxon Signed‐Rank test followed by Bonferroni correction.

**Results:**

The mean IRP with and without the RNS was 19.1 and 14.7 mm Hg, respectively (*p* < 0.001). Using the inflow port of the RNS for irrigation created a lower IRP (19.1 mm Hg) compared to the outflow port (32.7 mm Hg; *p* < 0.001). Addition of suction to all working scenarios significantly reduced IRP (*p* < 0.001). Insertion of instruments did not significantly alter IRP.

**Conclusion:**

In situations where the IRP should be low, removal of the RNS, irrigating through the inflow port, and frequent use of suction maintain the lowest pressures. If temporary increases in IRP are necessary to improve visualisation in the setting of bleeding, irrigating through the outflow port, minimising drainage and use of the RNS can be used to raise IRP.

## 1 INTRODUCTION

During percutaneous nephrolithotomy (PCNL), adequate intrarenal pressure (IRP) is important for preventing collapse of the pelvicalyceal system and facilitates visualisation of renal calculi and anatomical landmarks.^1^ However, an increase in IRP above 30 mm Hg has been linked to pyelovenous backflow, which may lead to postoperative fever, pyelonephritis and sepsis because of the systemic absorption of bacteria and endotoxins.^2^, ^3^, ^4^ Additionally, patients with elevated IRP have been shown to have greater postoperative pain and longer hospital stays, increasing both patient suffering and economic burden.^5^ Given the importance of managing IRP during PCNL, surgeons should understand factors within their control that may be employed to adapt the IRP to the clinical scenario encountered.

Previous studies have explored the impact of access sheath size,^3^ patient positioning,^6^ multiple tracts^7^ and different‐sized nephroscopes on IRP.^8^ Recently, surgeons have removed the outer metal sheath from the rigid nephroscope to reduce morbidity. However, the effect of removing the RNS on IRP has not been studied. In addition, there are many different factors that could affect IRP, including whether the inflow or outflow port is used for irrigation, the use of ancillary instruments like the two‐prong grasper (TPG) and ultrasonic lithotripter (USL), and the use of suction.

The purpose of this study was to evaluate the effect of using a rigid nephroscope with and without the outer metal sheath, irrigation through the inflow or outflow port, the use of different instrument combinations, and the presence or absence of suction on IRP in a benchtop PCNL model.

## 2 MATERIALS AND METHODS

**FIGURE 1:**
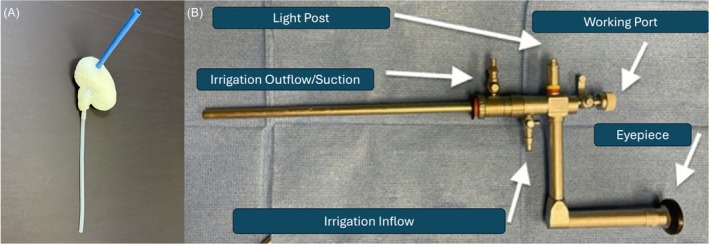
(A) Image of silicone kidney and ureter model. (B) Diagrammatic representation of rigid nephroscope used in the study demonstrating inflow and outflow ports.

A benchtop model was developed to simulate a PCNL procedure to evaluate how different instruments and irrigation combinations affect IRP. This model utilised anatomically representative kidney and torso models to simulate a patient in the prone position undergoing a PCNL. Three‐dimensional (3D) kidney models were obtained from a deidentified CT of a patient and processed using 3D slicer 5.2.2 (Kitware, NY).^9^ The source patient whose kidney was utilised for the 3D kidney models was chosen to represent an ‘average’ PCNL patient with normal anatomy and moderate hydronephrosis. The digital mould was printed using an Ultimaker 3 3D printer (Ultimaker, Utrecht, Netherlands) and then coated with liquid Dragon Skin™ 20 silicone (Smooth‐On, Inc., Macungie, PA). To simulate the ureter, a 30‐cm long, 4.3‐mm inner diameter polyvinyl chloride tube was attached to the renal hilum with silicone. Finally, the two halves of the silicone kidney model were sealed with additional silicone to create a water‐impermeable renal pelvis with calices (Figure 1).

To create the torso model, plaster rolls that were pre‐soaked in warm water were wrapped around a Laerdal patient mannequin torso (Laerdal Medical, Stavanger, Norway). The plaster was left to dry for 24 h. Measurements were taken from a prone patient CT scan to accurately position the kidney model within the torso. Utilising these measurements, wooden dowels were cut accordingly and fixed to the plaster shell and silicone kidney to accurately position the silicone kidney within the prone torso model. The remaining space within the torso model was filled with Big Gap Filler (Great Stuff, Austin, TX). Holes were carefully drilled through the torso and the kidney model to allow the placement of a 30‐Fr Amplatz renal sheath (Boston Scientific, Marlborough, MA) into the collecting system. The silicone kidney model and Amplatz sheath were then removed from the torso model, and the Dragon Skin™ was then applied around the Amplatz sheath to secure it to each kidney model with a water‐tight seal. BegoStone phantoms (Bego USA, Lincoln, RI), with a volume of 765 mm^3^, were placed within the renal pelvis to simulate fluid dynamics intraoperatively. A 7.95 Fr URF‐P6R flexible ureteroscope (Olympus, Tokyo, Japan) was placed with the distal tip inside the renal pelvis. An arterial line transducer and pressure monitor (Medical Data Electronics, Arleta, California) were employed to measure pressure in mm Hg.

Five trials were performed for each of the four endoscopic tool combinations that could influence IRP. The first variable tested was the presence or absence of the RNS on the 26 Fr nephroscope (KARL STORZ SE & Co. KG) (Figure 1). The second variable was whether the irrigation was connected to the inflow or the outflow port of the nephroscope. The third variable was the presence of different instruments introduced through the working channel of the nephroscope, including a 9.8 Fr TPG (Olympus Tokyo, Japan) or 3.7 mm USL (Olympus, Tokyo, Japan). The fourth variable was whether suction was used with the USL or not. Finally, IRP with the presence of a flexible nephroscope with and without laser was compared. All these variables were combined to create a total of 14 different working scenarios. In all scenarios, gravity overhead irrigation was utilised and set at 90 cm from the bottom of the bag to the position of the renal pelvis. A priori comparisons between specific configurations were conducted utilising the Wilcoxon Signed‐Rank test. The *p*‐values were adjusted utilising Bonferroni correction for multiple comparisons, with *p* < 0.05 considered significant.

## 3 RESULTS

**FIGURE 2:**
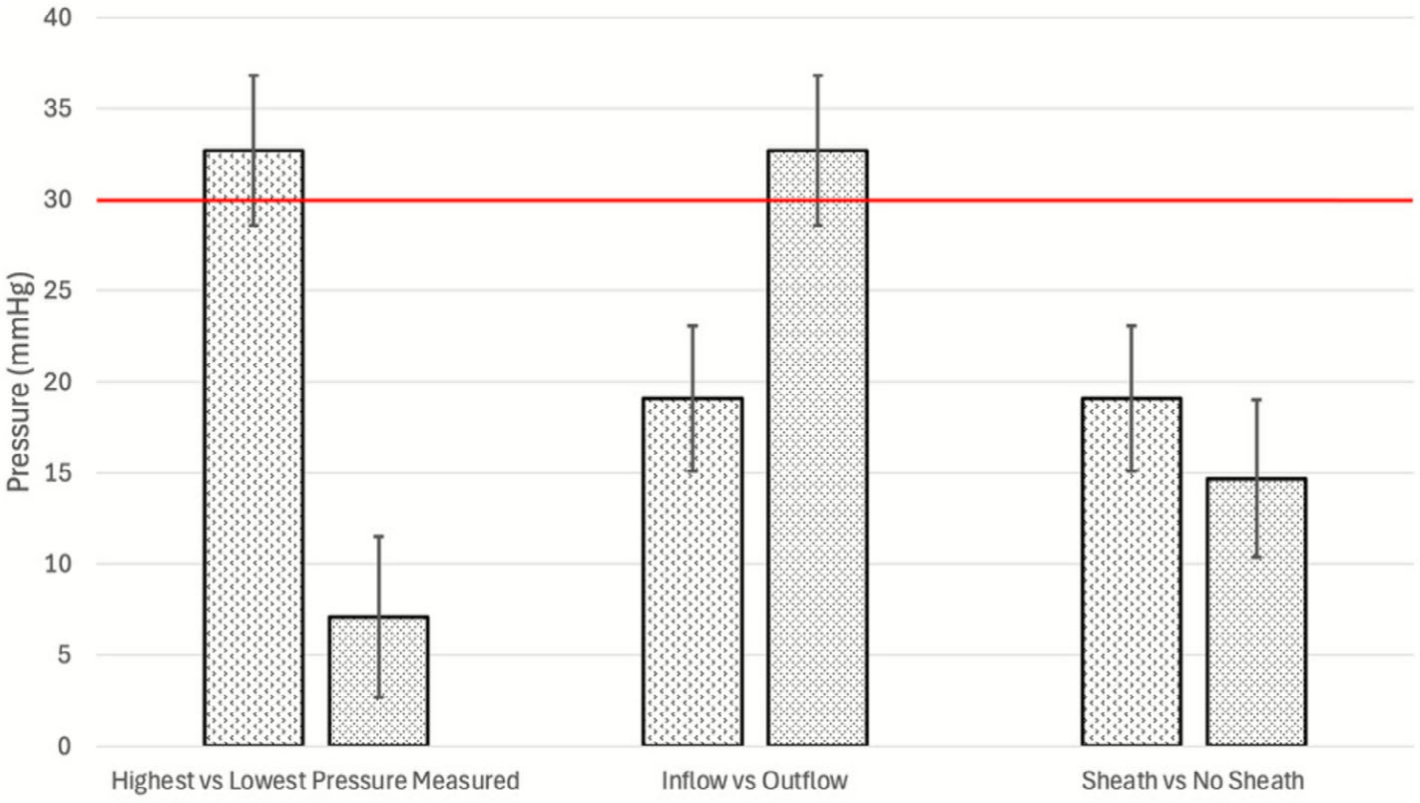
Demonstration of different IRPs based on the selected configuration (highest pressure measured vs lowest pressure measured, use of inflow vs outflow irrigation and RNS vs no RNS). IRP, intrarenal pressure; RNS, rigid nephroscope sheath.

Baseline IRP, obtained utilising the conventional nephroscopic configuration with the RNS and irrigation through the inflow port, recorded a mean pressure of 19.1 ± 4.0 mm Hg and did not cross the theoretical threshold of unsafe IRP (30 mm Hg) (Table 1). The highest IRP recorded was 32.7 ± 4.1 mm Hg with the RNS and outflow port irrigation, while the lowest IRP was 7.1 ± 4.4 mm Hg without RNS, inflow port irrigation and USL with suction (Figure 2).

**TABLE 1:** IRP comparison of variables tested.

Irrigation	Sheath	Accessory	Suction	Mean ± SD (mm Hg)
Inflow	Yes	None	No	19.1 ± 4.0
	No	None	No	14.7 ± 4.3
	Yes	USL	No	16.9 ± 5.1
	Yes	USL	Yes	7.8 ± 5.0
	Yes	TPG	No	18.9 ± 3.8
	No	TPG	No	15.0 ± 4.4
	No	USL	No	14.9 ± 4.7
	No	USL	Yes	7.1 ± 4.4
Outflow	Yes	None	No	32.7 ± 4.1
	Yes	TPG	No	30.4 ± 4.3
	Yes	USL	Yes	8.5 ± 4.5
	Yes	USL	No	29.7 ± 4.2

*Note*: IRP, intrarenal pressure; TPG, two‐prong grasper; USL, ultrasonic lithotripter.

Switching the irrigation from the inflow port to the outflow port increased the IRP by 71% from 19.1 ± 4.0 to 32.7 ± 4.1 mm Hg (*p* < 0.001; Figure 2). Inflow port irrigation with and without the RNS created an IRP of 19.1 ± 4.0 and 14.7 ± 4.3 mm Hg, respectively. Therefore, RNS use increased IRP by 30% (*p* < 0.001; Figure 2).

**FIGURE 3:**
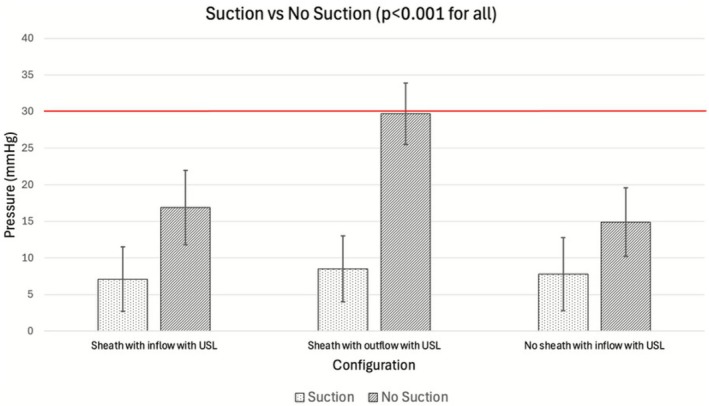
Demonstration of the effect of USL suction on IRP in different nephroscopic configurations. IRP, intrarenal pressure; USL, ultrasonic lithotripter.

Inserting the USL without RNS and using inflow port irrigation with or without the use of suction resulted in an IRP of 7.1 ± 4.4 and 14.9 ± 4.7 mm Hg, respectively. The addition of suction, removal of the RNS and use of inflow port irrigation reduced the IRP by 52% compared to no suction (*p* < 0.001; Table 2; Figure 3). Inserting the USL with the RNS and outflow port irrigation with and without the use of suction resulted in an IRP of 8.5 ± 4.5 and 29.7 ± 4.2 mm Hg, respectively. Adding suction to the RNS and outflow irrigation reduced the IRP by 71% (*p* < 0.001; Figure 3). The use of inflow port irrigation with and without the RNS recorded an IRP of 19.1 ± 4.0 and 14.7 ± 4.3 mm Hg, respectively. Excluding the RNS while using the inflow port irrigation reduced the IRP by 23% (*p* < 0.001; Figure 3).

**TABLE 2:** The significance of the difference between instrument configurations on IRP.

Comparison	*p*‐value
Irrigation through inflow: with vs without sheath	**<0.001**
With sheath: irrigation through outflow vs inflow	**<0.001**
With sheath and irrigation through outflow: with and without USL (no suction)	1.000
With sheath with USL and irrigation through outflow: with vs without suction	**<0.001**
With sheath and irrigation through outflow: with vs without TPG	1.000
Without sheath and irrigation through inflow: with vs without USL	1.000
Without sheath and irrigation through inflow: with vs without TPG	1.000
With sheath with USL and irrigation through inflow: with vs without suction	**<0.001**
Without sheath with USL and irrigation through inflow: with vs without suction	**<0.001**
Flexible ureteroscope with vs without laser	0.31

*Note*: Bold values indicate statistical significance.

Abbreviations: IRP, intrarenal pressure; TPG, two‐prong grasper; USL, ultrasonic lithotripter.

The IRP recorded during insertion of the TPG without the RNS was 15.0 ± 4.4 mm Hg, while the IRP for the USL without suction or the RNS was 14.9 ± 4.7 mm Hg. Use of the TPG with the RNS and inflow irrigation recorded an IRP of 18.9 ± 3.8 mm Hg, which is 26% higher than when operated without the RNS (*p* = 1.000). Employing the USL without suction using the inflow port and including the RNS recorded an IRP of 16.9 ± 5.1 mm Hg, which increased IRP by 13% compared to no use of the RNS (*p* = 1.000). The IRP recorded for the RNS and inflow port irrigation with and without the USL was 16.9 ± 5.1 and 19.1 ± 4.0 mm Hg, respectively. Removing the USL increased the IRP by 13% (*p* = 1.000). The IRP recorded for the RNS with and without the TPG was 18.9 ± 3.8 and 19.1 ± 4.0 mm Hg (*p* = 1.000), respectively. The addition of the TPG increased the IRP by 1% (*p* = 1.000). Finally, the insertion of a 16 Fr flexible nephoscope with or without a laser fibre, created an IRP of 13.6 ± 4.3 and 13.2 ± 4.6 mm Hg, respectively (*p* = 0.31).

## 4 DISCUSSION

In this experiment, IRP was simulated for prone PCNL by measuring 14 different combinations of RNS, irrigation attachment configurations and nephroscopic auxiliary tools. The highest IRP recorded was when combining the RNS and outflow port irrigation without the use of suction, and the lowest IRP recorded was when excluding the RNS and using inflow port irrigation and suction. Additionally, the use of nephroscopic tools such as a TPG or USL as well as a flexible nephroscope with or without laser did not significantly alter the IRP. Possible explanations for how each change of nephroscopic configuration altered the IRP are as follows: First, the use of the RNS created smaller diameter discrepancy with the Amplatz sheath, which made a narrow channel for irrigation fluid to escape around the nephroscope, resulting in a higher recorded IRP. Second, irrigating through the 4 Fr inflow port is routinely employed to provide good irrigation flow without overpressurising the system. However, irrigating through the outflow port which is larger (8 Fr) can be used in circumstances like when bleeding is encountered in non‐infected stones to reduce bleeding and improve visualisation. However, the opposite was not true, as when the outflow port was utilised, the drainage through the small inflow port led to a higher IRP. Finally, the use of suction had a significant impact on reducing IRP because of the rapid reduction in volume of irrigation.

Intrarenal pressure can have a significant effect upon the success of intrarenal stone surgery. Increased IRP may lead to pelvicalyceal system distention, which may improve intraoperative visualisation and facilitate the identification of intrarenal anatomy.^1^ However, persistent increase in IRP above 30 mm Hg for periods of 40–60 s has been associated with increased postoperative fever,^10^ pain and prolonged hospital stay.^5^ Hinman et al., while studying the effects of IRP in canine models, concluded that high IRPs (>30 mm Hg) were associated with damage to the renal filtration unit. The adverse events occurring with high IRP were found to be because of the direct transmission of IRP to the nephrons and the backflow of fluids into the systemic circulation and surrounding tissues.^11^ High pressure in the renal pelvis is transmitted to the minor calyx, in the direction of the medulla. This pressure compresses the peritubular microvasculatures, causing ischaemia and loss of the filtration unit. Another mechanism of injury exerted by high IRP is intrarenal fluid backflow through the pyelovenous, pyelosinus, pyelolymphatic, pyelotubular and pyelointerstitial routes. This backflow gives access for bacteria and toxins in urine as well as irrigation fluid to enter the systemic circulation and kidney parenchyma, leading to sepsis, electrolyte imbalance and fluid overload.^12^ The IRP threshold for fluid backflow is not clear. However, it was found that even at an IRP lower than 30 mm Hg, infected kidneys are subjected to septic complications.^13^ The renal pelvis is known to be compliant; however, excessive IRP may cause rupture and urine leak,^14^ leading to abdominal and fluid collections, urinary peritonitis, abscesses or fistulas.^15^

A logical starting point, therefore, is to adopt a strategy that maintains low IRP during PCNL; however, this is not the case in all operative scenarios. Inadequate distention of the collecting system compromises visibility, risking urothelial injuries.^7^ Elevated IRP normally controls minor venous bleeding via the tamponade effect; however, when IRP is low, as is the case in multi‐track procedures^7^ or in the supine position,^6^ it can lead to increased bleeding that can obscure the field, potentially prolonging the operative time. Furthermore, low IRP can hinder stone clearance by reducing fragment efflux and increasing the risk of residual fragments leading to postoperative pain, obstruction and need for additional procedures.^16^

The concept of an optimal IRP remains elusive, as no single pressure suits all patients or surgical situations. IRP requirements vary depending on the individual anatomy, stone complexity and procedural factors. This presents a recurring intraoperative challenge: whether to lower IRP to minimise pyelovenous backflow and sepsis risk at the cost of poor visibility, or to raise IRP to improve visibility, reduce bleeding, and enhance stone clearance, that is, the Goldilocks dilemma.^17^ Thus, a solid understanding of IRP dynamics and the variables influencing it is essential, allowing surgeons to use IRP as a modifiable parameter to tailor operative strategies case by case.

Based on this experiment and our experience in intrarenal stone surgery, we propose the use of different nephroscopic configurations, irrigation sources and instruments depending on the intraoperative challenges. For infected systems like staghorn, fungal or matrix stones, using inflow port irrigation, intermittent suction, and removal of the RNS can lower IRP, reducing sepsis risk and improving visibility. In contrast, for recurrent, non‐infected hard stones like those in cystinuria or primary hyperoxaluria, an intermittent high IRP approach using the RNS, outflow port irrigation and reduced suction can improve visibility by controlling bleeding and enhancing stone clearance. In addition to the variables tested, Farkouh et al. found that performing PCNL in the supine position has been shown to result in lower IRP compared to the prone position and serves as another strategy for lowering intrarenal pressure.^6^

This study has its limitations. First, using a benchtop model, it is difficult to simulate real‐time human tissue factors such as different anatomical variabilities, tissue compliance, vascularity and fluid–tissue exchange dynamics. However, this model enabled controlled, replicable comparisons between trials. Additionally, the model was created from a single patient's CT scan. As noted by Chen et al., prior endourological surgery and anatomical variations can significantly influence IRP.^18^ The utilisation of a single 30 Fr access sheath provided us with an adequate understanding of the relationship between IRP and different nephroscopic configurations and tool use. However, future investigations could explore the effects of different access sheath calibres in relation to different sized instrumentation such as balloon dilators, baskets and various laser fibres. The addition of different types and sizes of instruments and the investigation of the effect of retrograde drainage in combination with the studied configurations are warranted. Additionally, while we measured peak pressure changes, we did not assess the duration of elevated pressures. Longer durations of pressure elevation may potentially be more damaging.^4^ Another limitation of this study is the exclusive use of a standard nephroscope. This design was chosen to isolate the effects of instrumentation and irrigation and to avoid replication of extensive prior research comparing the standard versus mini or ultra‐mini PCNL techniques.^3^, ^19^, ^20^ One of the benefits of using the standard 30 Fr sheath is the presence of these modifiable factors that can be used to optimise intrarenal pressure to the unique anatomical and physiological clinical scenario and patient needs. These same modifiable factors are not present in mini and ultra‐mini PCNL, and subsequently this research is not generalisable to these smaller access strategies. The focus on standard PCNL remains clinically relevant, as it is still widely used for patients with a high stone burden and may have shorter operative times in these patients.^19^ Finally, implementing various pressure monitoring and adjustment systems would enhance the understanding of the IRP and allow it to be used as a tool rather than a negative variable.

## 5 CONCLUSION

This study demonstrates the role of inflow and outflow management in controlling IRP during PCNL. Strategies to ensure adequate outflow, such as avoiding the rigid nephroscope sheath use, irrigation through the inflow port and use of active suction can significantly reduce IRP and, therefore, potentially decrease the risk of pressure‐related complications. When selecting accessories, the surgeon should use the one that is most effective because the instruments tested had no significant effect on IRP. Knowledge of these variables that can influence IRP can be customised by the surgeon to lower the surgical risk and improve surgical outcomes.

## CONFLICT OF INTEREST STATEMENT

The authors declare no conflicts of interest.
